# The use of simultaneous stereo-electroencephalography and magnetoencephalography in localizing the epileptogenic focus in refractory focal epilepsy

**DOI:** 10.1093/braincomms/fcab072

**Published:** 2021-04-08

**Authors:** Umesh Vivekananda, Chunyan Cao, Wei Liu, Jing Zhang, Fergus Rugg-Gunn, Matthew C Walker, Vladimir Litvak, Bomin Sun, Shikun Zhan

**Affiliations:** 1 Department of Clinical and Experimental Epilepsy, UCL, Queen Square Institute of Neurology, London WC1N 3BG, UK; 2 Department of Neurosurgery, Ruijin Hospital, Shanghai Jiao Tong University, School of Medicine, Shanghai 200025, China; 3 Wellcome Centre for Human Neuroimaging, UCL, Queen Square, London WC1N 3AR, UK

**Keywords:** magnetoencephalography, stereo-electroencephalography, epilepsy surgery

## Abstract

Both magnetoencephalography and stereo-electroencephalography are used in presurgical epilepsy assessment, with contrasting advantages and limitations. It is not known whether simultaneous stereo-electroencephalography–magnetoencephalography recording confers an advantage over both individual modalities, in particular whether magnetoencephalography can provide spatial context to epileptiform activity seen on stereo-electroencephalography. Twenty-four adult and paediatric patients who underwent stereo-electroencephalography study for pre-surgical evaluation of drug-resistant focal epilepsy, were recorded using simultaneous stereo-electroencephalography–magnetoencephalography, of which 14 had abnormal interictal activity during recording. The 14 patients were divided into two groups; those with detected superficial (*n* = 7) and deep (*n* = 7) brain interictal activity. Interictal spikes were independently identified in stereo-electroencephalography and magnetoencephalography. Magnetoencephalography dipoles were derived using a distributed inverse method. There was no significant difference between stereo-electroencephalography and magnetoencephalography in detecting superficial spikes (*P* = 0.135) and stereo-electroencephalography was significantly better at detecting deep spikes (*P* = 0.002). Mean distance across patients between stereo-electroencephalography channel with highest average spike amplitude and magnetoencephalography dipole was 20.7 ± 4.4 mm. for superficial sources, and 17.8 ± 3.7 mm. for deep sources, even though for some of the latter (*n* = 4) no magnetoencephalography spikes were detected and magnetoencephalography dipole was fitted to a stereo-electroencephalography interictal activity triggered average. Removal of magnetoencephalography dipole was associated with 1 year seizure freedom in 6/7 patients with superficial source, and 5/6 patients with deep source. Although stereo-electroencephalography has greater sensitivity in identifying interictal activity from deeper sources, a magnetoencephalography source can be localized using stereo-electroencephalography information, thereby providing useful whole brain context to stereo-electroencephalography and potential role in epilepsy surgery planning.

## Teaser

In the largest case series to date, Vivekananda et al. perform simultaneous magnetoencephalography and stereo-electroencephalography in refractory focal epilepsy patients. They demonstrate that for deep brain epileptogenic foci, magnetoencephalography dipoles could be derived from average stereo-electroencephalography data, thereby overcoming a traditional limitation of magnetoencephalography of reduced sensitivity for deep sources.

## Graphical Abstract

**Graphical Abstract:**
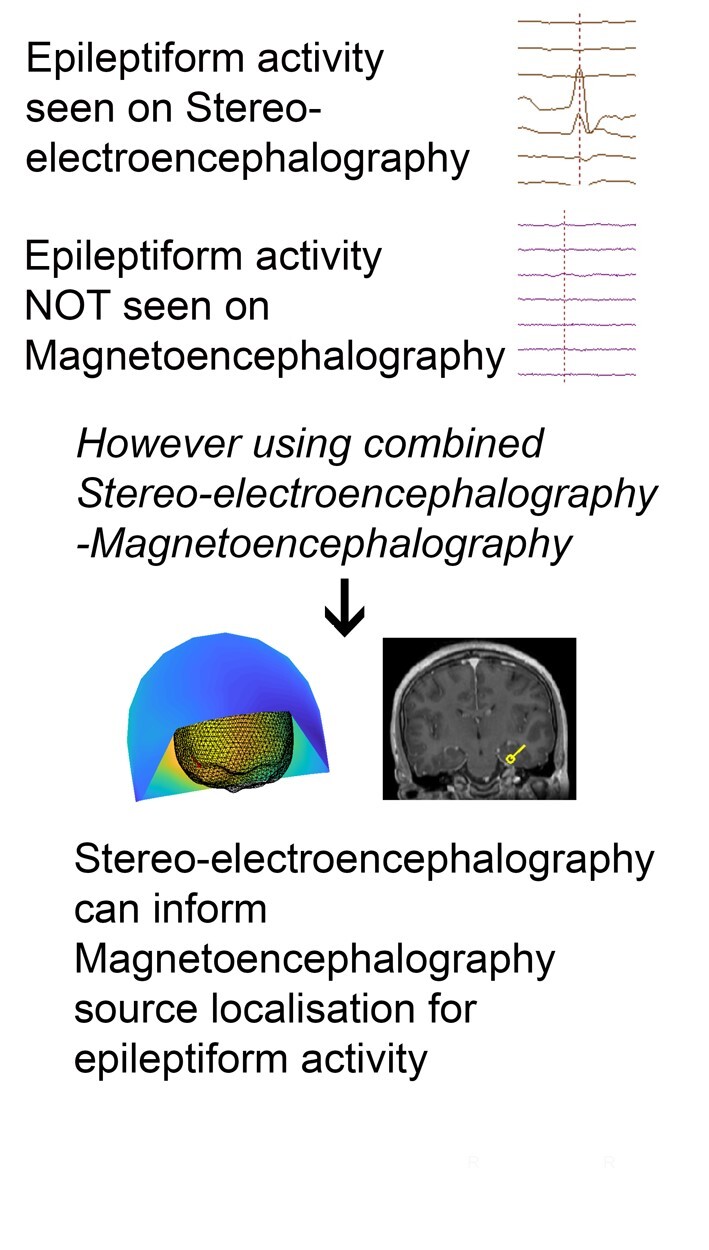

## Introduction

Magnetoencephalography (MEG) and stereo-electroencephalography (SEEG) can provide complementary information for the presurgical assessment of refractory focal epilepsy. MEG is non-invasive, has high temporal and spatial resolution with good global coverage, and unlike surface electroencephalography is not affected by skull conductivity.^1^^,^^2^ However, deep sources such as the mesial temporal lobe, a region commonly associated with refractory epilepsy, are poorly detected with MEG, meaning its use in such cases is of less value.^3–5^ This is likely because the spatial resolution decreases rapidly as a function of the depth of the epileptic generators, making source estimation challenging.^6^ MEG is also insensitive to radially orientated sources, e.g. surface of a cortical gyrus.^7^ It has also been shown that physiological deep brain activities can be detected using MEG if informed by SEEG^8^, although its relevance in a clinical context is uncertain, i.e. can MEG informed by SEEG demonstrate epileptiform activity previously not demonstrated using MEG alone. SEEG is an invasive procedure, in which a limited set of electrodes are placed within the brain; these provide excellent detection of adjacent sources but have restricted spatial sampling. Therefore, it is difficult to interpret whether the epileptogenic zone (EZ) and SEEG electrode contacts identifying the abnormal epileptiform activity truly co-localize, or whether the zone is actually situated in nearby functionally connected brain structures. This is one of a number of reasons, including underlying pathology, age at time of surgery, and brain region where EZ was located (extra-temporal versus temporal), that surgical resection of the EZ as identified by SEEG results in a 60–70% chance of achieving seizure freedom.^9^ MEG has previously been compared with SEEG non-concurrently, demonstrating that concordance between both modalities in identifying epileptiform activity was associated with a higher chance of seizure freedom post resection.^10^^,^^11^ However, these studies acknowledged the limitation that MEG recordings are necessarily brief (∼1 h) compared with SEEG telemetry over several days, leading to uncertainty in whether interictal activity captured by MEG and SEEG relate to identical epileptogenic foci. This is particularly relevant for scenarios where magnetic source imaging produces dispersed MEG dipoles,^11^ which are difficult to interpret and require SEEG confirmation of the EZ. Simultaneous SEEG–MEG recording should be able to resolve such questions.

Few clinical studies on single cases have reported simultaneous SEEG and MEG recordings, most likely due to technical challenges in its acquisition.^12–14^ We hypothesize the following; interictal activity identified by MEG and SEEG relate to the same epileptogenic focus, simultaneous SEEG–MEG can improve the identification and localization of deep brain epileptogenic sources compared to either modality alone, and removal of this source relates to good post epilepsy surgery outcome.

## Materials and methods

### Simultaneous SEEG–MEG recordings

Patients (adults and children) who underwent SEEG study for pre-surgical evaluation of drug-resistant focal epilepsy at Shanghai Jiaotong University School of Medicine during 2017 and 2019 were considered for the study. Every patient was informed about the aim and the scope of the study and gave written informed consent. Implantation of intracranial electrodes (SDE-08: S8, S16, Beijing Sinovation Medical Technology CO., LTD, Beijing, China) was under general anaesthesia with planning guided by clinical indications, informed by prior MRI (results in Table 1), video-electroencephalography, MEG and PET studies. Of this cohort, patients who had 8 or less implanted SEEG electrodes were included for the study, due to the space constraint of the MEG helmet for simultaneous recording. Therefore, 24 patients who met this inclusion criteria were subsequently analysed. Two types of electrodes were used over the whole patient group; for electrodes with 8 contacts, the deepest contact is named as 1 and the most superficial contact named 8. For electrodes with 16 contacts, the deepest channel was named as 1 and the most superficial contact named 16. The length of contact was 2 mm, the distance between contact was 1.5 mm and the diameter of electrode was 0.8 mm.

**Table 1:** Demographics and SEEG implantation for epilepsy patients included in study

Case of Patient	Age (ys)	Gender	Implantation areas and (number of electrode/contacts)	Pre-operative MRI
1	47	F	Right T, P (4/32)	Previous left T meningioma removal
2	14	F	Right F, T, P (4/32)	No abnormality
3	33	F	Left T, P (4/32)	Left HPC sclerosis
4	18	F	Right F, T, and Left F and O (4/32)	Minimal left HPC atrophy
5	26	F	Left T, insular, O, right T (5/48)	No abnormality
6	27	F	Left F, P, T (4/32)	Minimal left HPC atrophy
7	23	F	Left F, T, P and Right T (5/40)	No abnormality
8	19	M	Left T, P, O, and Right T, P (6/48)	Abnormal left P-O signal
9	26	M	Right F, T, P (4/32)	No abnormality
10	44	M	Left F, T, P (4/32)	No abnormality
11	9	F	Left T, O and Right T (6/64)	Abnormal left O signal
12	32	M	Left F, T and P (8/64)	Left HPC sclerosis
13	27	F	Left T, O (8/64)	Left HPC atrophy
14	12	F	Right F, T, P and left T (7/60)	No abnormality

F, frontal; HPC, hippocampus; O, occipital; P, parietal; T, temporal.

Location and number of SEEG electrodes implanted varied between patients depending on detected epileptogenic focus (Table 1).

Simultaneous ∼7 min SEEG–MEG recordings were performed with patient sitting upright using a 306-channel, whole-head VectorView MEG system (Elekta Oy, Helsinki, Finland) in a magnetically shielded room (Euroshield, Eura, Finland) situated within the hospital epilepsy unit. A clinician and scientist were present throughout the recording for patient safety. Light head bandaging was used and SEEG cap replaced if felt necessary after recording; no infection was reported in these patients. For SEEG, cable length from the head to the connectors was about 50 cm and the amplifier was powered via an isolated 24 V transformer situated in a separate electronics cabinet.

Raw MEG data were band pass filtered 0.03–330 Hz and digitized at 1000 Hz. The magnetic artefacts and movement artefact were removed by the temporal extension of Signal Space Separation method (tSSS) implemented in the MaxFilter software (Neuromag 3.4, Elekta Oy, Helsinki, Finland). Ten patients had no epileptiform activity recorded on SEEG and so were excluded from further analysis. One patient had a seizure during recording.

SEEG analysis was performed using Brain Electrical Source Analysis software (BESA GmbH, Germany, http://www.besa.de/ last accessed 15 April 2021), Statistical Parametric Mapping (SPM12, UCL, https://www.fil.ion.ucl.ac.uk/spm/ last accessed 15 April 2021) and Fieldtrip (http://fieldtriptoolbox.org last accessed 15 April 2021). SEEG was analysed using bipolar montage (Band-pass filter 1–70 Hz, 50 Hz notch). Interictal spikes were visualized by two experts (U.V. and M.C.W) and manually marked using BESA software at the peak of maximal positive/negative deflection of the spike. The electrode contact with the largest average spike amplitude was then noted (annotated as ‘peak amplitude’ channel). Patients were then divided into superficial source and deep source groups dependent on the location of peak amplitude channel (deep: 1–3 contact number; superficial: 6–8 or 14–16 contact number). Data from all other contacts were disregarded as situated within white matter.

**Figure 1:**
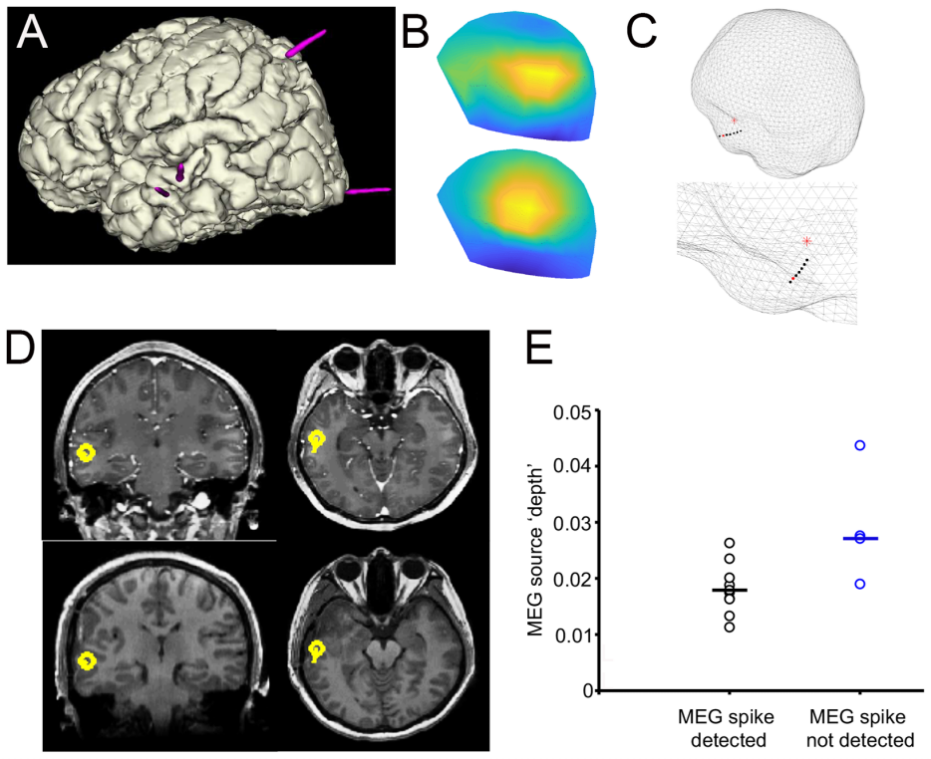
**Example patient with superficial epileptogenic focus (Pt 5)**. (**A**) Schematic of electrode placement. (**B**) Field map (measured top and modelled bottom) corresponding to the peak of the average interictal spike. (**C**) Relation on inner skull mesh of MEG dipole (red star) and electrode contact (red circle) with highest spike amplitude, top, and zoomed region, below. (**D**) Upper panel is the position of MEG dipole on pre-operative MRI scan coronal and sagittal planes; lower panel is post-operative MRI scan. (**E**) Relationship within patients between MEG source ‘depth’ and presence of MEG spikes, horizontal bar indicating mean depth.

Locations of implanted SEEG electrodes were identified from postoperative CT scans using Lead-DBS toolbox (https://www.lead-dbs.org/ last accessed 15 April 2021) (Figs. 1A and 3A). Post-operative CT was co-registered with a pre-operative T1 structural MRI in SPM12 and further adjusted under manual control using Slicer software (https://www.slicer.org/ last accessed 15 April 2021). SEEG contact locations were then obtained by manually fitting electrode models to the artefacts seen in the CT, with white matter contacts rejected, using the interface implemented in Lead-DBS.

**Figure 2:**
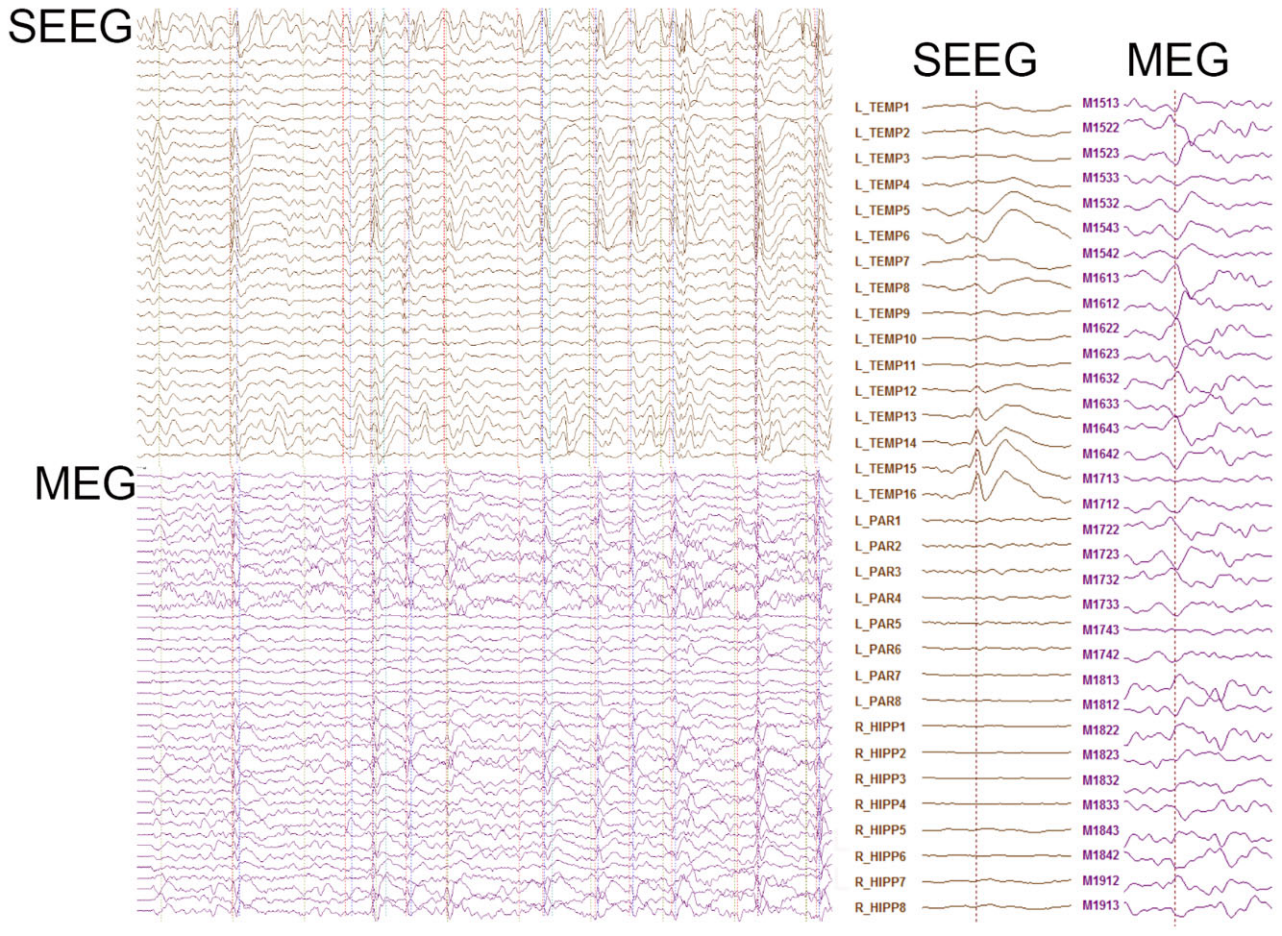
**Example SEEG and MEG traces from Pt 5.** Raw SEEG (top) and MEG (bottom) traces with dotted lines indicating marked spikes, and magnified average SEEG and MEG interictal spike (right).

Analysis of the MEG recording was performed ‘blind’ to SEEG findings. Interictal spikes were identified using BESA software (Band-pass filter 1–35 Hz, 50 Hz notch, gain 400–800 fT). All spikes were manually marked by two experts (U.V. and F.R.-G.) at the peak of maximal positive deflection of the spike.

Source localization for MEG data was performed by averaging individual spikes (BESA software), before importing into SPM. A time window was then set 100 ms before the rise phase and 100 ms after the fall phase of the average spike. A single shell forward model^15^ based on canonical meshes inverse normalized^16^ to the pre-operative T1 structural MRI image was used, thereby creating an individual model for each patient. A single MEG dipole (Fieldtrip) was fitted and the corresponding residual variance image was also examined, in order to easily calculate distance between MEG and SEEG sources. A three dimensional co-ordinate in native space was found for both SEEG peak amplitude channel and MEG dipole, in order to calculate this distance. If there were no spikes seen on MEG alone, MEG source activity (M-source) was derived by averaging the raw MEG data informed by co-existent SEEG spikes (taken 1 s before and after highest amplitude of spike), before the same dipole fitting process was performed. This was in order to test if SEEG–MEG simultaneous recording could provide more information than visual inspection alone. To examine any relationship between ‘depth’ of M-source and presence of MEG spikes, we calculated the distance (d) between the anterior commissure (AC) and dipole location in MNI space, and used 1/d as an estimated measure of ‘depth’. Where possible, post-resection MRI images were co-registered to the pre-operative T1 structural MRI to confirm whether M-source location was removed during surgery, and then related this to surgery outcome.

Number of spikes identified by SEEG and MEG were compared using a paired *t*-test for superficial brain sources and Mann–Whitney U-test for deep brain sources. M-source ‘depth’ and presence of MEG spikes was compared using *t*-test.

### Ethics approval

The study was approved by the local ethics committee of Shanghai JiaoTong University.

### Data availability

Anonymized data will be shared by request from any qualified investigator.

## Results

We first manually identified interictal spikes separately for SEEG and MEG during the simultaneous recording and grouped for each patient number of spikes occurring in SEEG alone, occurring concurrently in SEEG and MEG and occurring in MEG alone (Table 2). For patients whose detected epileptogenic focus was superficial cortex, as defined by the SEEG peak amplitude occurring in an electrode contact greater than four, we found no statistical difference between number of spikes identified by SEEG versus MEG (paired *t*-test, *P* = 0.135) (Fig. 2). This suggests that SEEG and MEG are equally sensitive in identifying interictal spikes from cortical sources. For patients with a detected deep brain epileptogenic focus defined by SEEG peak amplitude occurring in an electrode contact smaller than three, we found that number of spikes identified by SEEG was significantly higher than MEG (Mann–Whitney U-test, *T* = 56.5, *P* = 0.002) (Fig. 4), indicating that SEEG is more sensitive than MEG in identifying interictal spikes from deep sources (Table 2).

**Figure 3:**
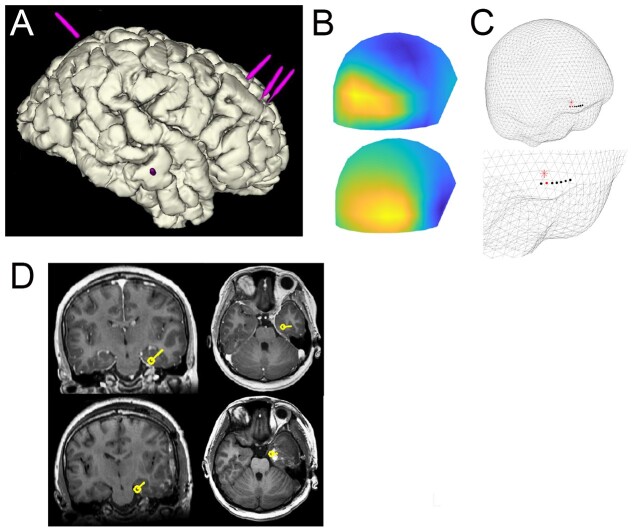
**Example patient with deep epileptogenic focus (Pt 14).** (**A**) Schematic of electrode placement. (**B**) Field map corresponding to the peak of the average epileptic spike. (**C**) Relation of MEG dipole and electrode contact with highest spike amplitude. (**D**) Position of MEG dipole on pre-operative MRI (upper) and post-operative MRI (lower).

**Table 2:** Number of interictal spikes identified on SEEG alone, SEEG and MEG both, and MEG alone for detected superficial and deep brain sources

Source	Region	Case of patient	Quantity of identified spikes			‘Peak amplitude’ channel	MEG dipole and ‘peak amplitude’ distance (mm)
			SEEG alone	SEEG and MEG co-incidentally	MEG alone		
Superficial	Frontal	2	5	18	3	Right F 6–7	8.10
	Temporal	3	10	6	0	Left T 6–7	17.4
		4	4	11	4	Right T 6–7	28.8
		5	1	14	0	Left T 10–11	9.09
		10	5	17	3	Left T 7–8	20.7
		11	0	4	2	Left T 9–10	42.1
	Parietal	6	0	21	0	Left P 6–7	19.1
Deep	Temporal	1	22	1	0	Left HPC 2–3	11.3
		7	26	0	0	Right HPC 1–2	29.8
		9	7	4	1	Left HPC 1–2	21.2
		12	34	0	0	Left HPC 1–2	8.49
		13	26	0	0	Left HPC 2–3	13.9
		14	30	0	0	Right HPC 2–3	8.40
	Parietal	8	4	1	0	Left P 1–2	31.8

‘Peak amplitude’ channel indicates SEEG channel with highest amplitude average spike. F, frontal; HPC, hippocampus; O, occipital; P, parietal; T, temporal.

We next examined the relationship of MEG source activity (M-source) location, and location of SEEG peak amplitude channel. For patients with detected superficial epileptogenic regions (*n* = 7), the mean distance between source M-source and ‘peak amplitude’ channel in native space was 20.7 ± 4.4 mm. (Table 2), suggesting that location of average M-source was closely related to average SEEG interictal spike location for superficial epileptogenic regions (Fig. 1B and C). In two patients (Pt4 and 11), although SEEG identified interictal activity in the lateral temporal lobe during simultaneous recording, subsequent SEEG assessment of seizures located the seizure onset zone to be in frontal and occipital brain regions respectively. However, unlike SEEG, MEG during simultaneous recording accurately source localized to these regions (Supplementary Fig. 1).

In all superficial source patients, MEG dipole location was concordant with detected EZ on ictal SEEG and removed brain region during epilepsy surgery (Table 3), confirmed by post-operative MRI in three patients (Fig. 1D). In six out of seven cases (except patient 6), there was an Engel Class 1 outcome after 12 months.

**Table 3:** Post surgery outcomes after 1 year

Patient	SEEG (entire recording)	SEEG (simultaneous recording)	MEG dipole	Source location	Resection	Engel class
1	Left F and mesial T	Left mesial T	Left mesial T	Deep—HPC	Left T resection	I
2	Right F	Right F	Right F	Superficial—insular	Right F, insular, operculum resection	I
3	Left T	Left T	Left T	Superficial—temporal neocortex	Left T resection	I
4	Left F	Right T	Left F	Superficial—superior frontal	Right F resection	I
5	Left T	Left T	Left T	Superficial—temporal neocortex	Left T resection	I
6	Left P	Left P	Left P	Superficial—parietal cortex	Left P resection	II
7	Bilateral mesial T—left predominant	Right mesial T	Right mesial T	Deep—HPC	Left T resection	II
8	Left T and P	Left P	Left P	Deep—parietal	Left T resection	II
9	Left T and P	Left mesial T	Left P	Deep—parietal	Left P resection	I
10	Right T	Right mesial T	Right T	Superficial—temporal neocortex	Right T resection	I
11	Left T	Left T	Left T	Superficial—temporal neocortex	Left O resection	I
12	Left HPC	Left mesial T	Left mesial T	Deep—HPC	Left T resection	I
13	Left mesial T	Left mesial T	Left mesial T	Deep—HPC	Left T resection	I
14	Right mesial T	Right mesial T	Right mesial T	Deep—HPC	Right T resection	I

SEEG entire recording describes location derived from prolonged telemetry and SEEG simultaneous recording describes location derived from simultaneous SEEG/MEG recording. F, frontal; HPC, hippocampus; O, occipital; P, parietal; T, temporal.

In one patient (Pt 2), a seizure lasting 15 s was recorded. Source localization was performed on the first one second of high beta activity recorded at seizure onset, again using M-source and SEEG peak amplitude channel (Supplementary Fig. 2). The results were concordant with interictal findings.

**Figure 4:**
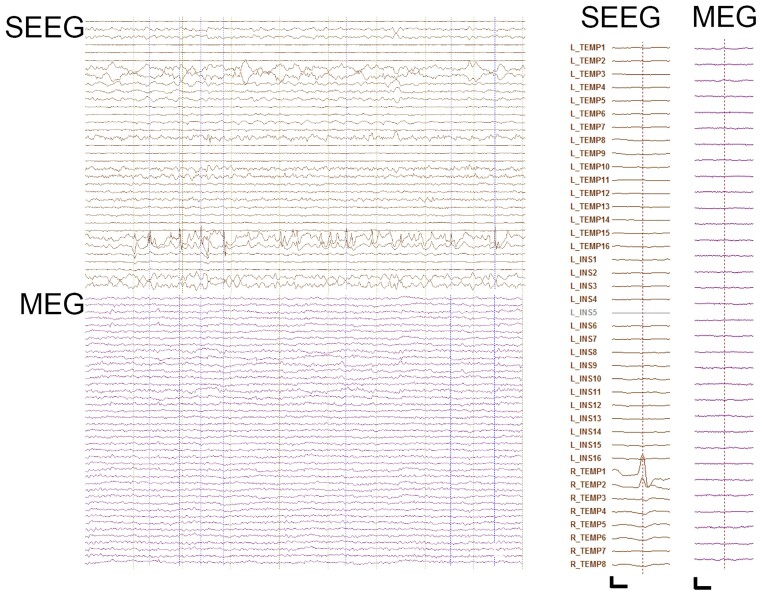
**Example SEEG and MEG traces from Pt 14**. Raw SEEG (top) and MEG (bottom) traces with magnified average SEEG and MEG interictal spike (right), Scale bar: 300 μV/500 fT and 0.2 s.

For patients with detected deep epileptogenic regions (*n* = 7), the mean distance between M-source and ‘peak amplitude’ channel was 17.8 ± 3.7 mm. (Table 2). In four mesial temporal cases (Pt 7,12,13,14), spikes were not visible to visual inspection on MEG, meaning that M-source was informed from SEEG spikes instead (Fig. 4). Interestingly even in these cases location of M-source was closely related to ‘peak amplitude’ channel location (Fig. 3B and C), suggesting that simultaneous MEG and SEEG have complementary localizing value, even in cases where no apparent MEG interictal activity is seen. There was a significant difference in MEG source depth between patients where MEG spikes were detected (mean 1/d from anterior commissure 0.0179 mm^−1^, *n* = 10) and not detected (0.0275 mm^−1^, *n* = 4), [*t*-test: *t*(8) = −2.5, *P* = 0.027; Fig. 1E].

In six out of seven patients, MEG dipole location was concordant with detected EZ on ictal SEEG and the removed brain region (Table 3), confirmed by post-operative MRI in three patients (Fig. 3D). In five out of the seven patients (except Patients 7 and 8), there was an Engel Class 1 outcome. The MEG dipole location in patient 8 (parietal lobe) was presumably outside of the resected brain region (temporal lobe), possibly explaining the persistence of seizures.

## Discussion

In the largest case series to date of epilepsy patients undergoing simultaneous SEEG–MEG study, we could directly compare the sensitivity for both modalities in identifying interictal spikes and spike localization. Previous studies have indirectly compared MEG and SEEG,^11^^,^^17^ with recordings performed at different time points (typically separated by a number of months) and therefore limited by variability in brain anatomy, disease status and medications taken between recordings. In addition, it is not always certain that the source of interictal activity recorded non-invasively with short-duration MEG recordings is identical to that identified using SEEG telemetry over several days. Here we could assess the same pathological epileptic brain discharges at both a local (SEEG) and global (MEG) level. We found that SEEG and MEG were comparable in identifying interictal spikes originating from superficial cortex, with MEG identifying spikes not viewed on SEEG in a number of patients. This likely reflects the relative limited spatial sampling SEEG provided. This finding however may be affected by the inherent bias that SEEG electrode placement is in part informed by prior MEG results, therefore an overlap of epileptogenic source identification between simultaneous SEEG and MEG would be expected. Average MEG dipole location was consistent with entire SEEG study findings in all superficial cases, and its removal during surgery was associated with an Engel class 1 outcome at 12 months in 6 out of 7 cases. In contrast, SEEG was superior in identifying spikes originating from key deep brain regions (e.g. mesial temporal). This has generally been perceived as a weakness of MEG both in clinical evaluation and in normal physiological studies, as it is known that spatial resolution in MEG is inversely proportional to source depth and complex deep structures such as the hippocampus can produce limited signal.^18^ However, extracting MEG information for deep brain epileptogenic regions is of importance as previous studies using separate SEEG and MEG recording have shown that anatomical correlation of MEG/SEEG led to a 66–85% chance of seizure freedom post epilepsy surgery compared with 11–30% when MEG/SEEG is discordant.^19^^,^^20^

We demonstrate a distance between average MEG dipole and peak amplitude SEEG channel of around 20 mm. for both superficial and deep epileptogenic sources. The reasons for this are probably multifactorial including propagation of interictal activity, limited spatial sampling of SEEG, and choice of MEG head model. Importantly, although spikes were not visible to visual inspection on MEG for the majority of patients with deep sources, average MEG activity informed by identified SEEG spikes still accurately localized deep source activity, which has not been demonstrated in epilepsy before. The likely reason for this finding was that averaging the MEG activity improved the low signal to noise ratio associated with deep brain activity. Our finding using a distributed inverse method commonly adopted in clinical MEG is consistent with the recent observation that SEEG informed deep brain MEG activity can be detected using independent component analysis.^8^ This has immediate implications for the use of simultaneous SEEG–MEG recording in epilepsy surgery planning, in terms of providing MEG spatial context to SEEG ictal and interictal activity. Further work would involve performing independent component analyses on SEEG informed MEG data, in order to in future detect and localize deep source abnormal epileptiform activity not evident on visual inspection when using non-invasive MEG recording alone. Another direction would be further imaging of seizures to provide more detailed spatial information on seizure propagation, and epileptic networks involved. We further show that surgical resection of the consequent average MEG dipole predicted seizure freedom at 12 months (5 out of 6 patients); the one patient where the dipole was not removed had seizure recurrence. However, more subjects would be required to properly assess the utility of SEEG–MEG recordings in predicting outcome post epilepsy surgery.

There were limitations of the study, including the brevity of recordings and consequently the limited number of cases analysed, which was dictated by the technical difficulty of acquiring simultaneous SEEG and MEG data. Secondly, due to the size constraint of the MEG helmet, patients selected for simultaneous recording had a maximum of 8 implanted SEEG electrodes. Therefore, the relatively low SEEG coverage may have influenced direct comparison of SEEG to MEG for detection of spikes. However, one may argue that denser SEEG coverage would have further improved SEEG informed MEG deep brain activity localization due to increased information. Further development of this method would involve longer recording times and accommodation of larger SEEG implantation schemes when recording MEG.

Simultaneous SEEG and MEG can thus provide complementary information about the spatial extent of interictal epileptiform activity, in particular for deep epileptogenic sources, and so better inform resection planning.

## Supplementary material

Supplementary material is available at *Brain Communications* online.

## Supplementary Material

fcab072_Supplementary_Data

## Abbreviations

EZ =: epileptogenic zone
MEG =: magnetoencephalography
SEEG =: stereo-electroencephalography
