# Erratum to: The use of simultaneous stereo-electroencephalography and magnetoencephalography in localizing the epileptogenic focus in refractory focal epilepsy

**DOI:** 10.1093/braincomms/fcab276

**Published:** 2021-11-30

Umesh Vivekananda, Chunyan Cao, Wei Liu, Jing Zhang, Fergus Rugg-Gunn, Matthew C. Walker, Vladimir Litvak, Bomin Sun and Shikun Zhan; The use of simultaneous stereo-electroencephalography and magnetoencephalography in localizing the epileptogenic focus in refractory focal epilepsy. *Brain Communications* 2021. doi: https://doi.org/10.1093/braincomms/fcab072.

In the originally published version of this manuscript, the graphical abstract was omitted. The publisher apologizes for the error.

