# Supplementary material for: The use of simultaneous stereo-electroencephalography and magnetoencephalography in localizing the epileptogenic focus in refractory focal epilepsy
Source: Brain Commun. 2021 Apr 8;3(2):fcab072. doi: 10.1093/braincomms/fcab072 (PMC8099997; doi:10.1093/braincomms/fcab072)
Supplement: fcab072_Supplementary_Data [file fcab072_supplementary_data.pdf]

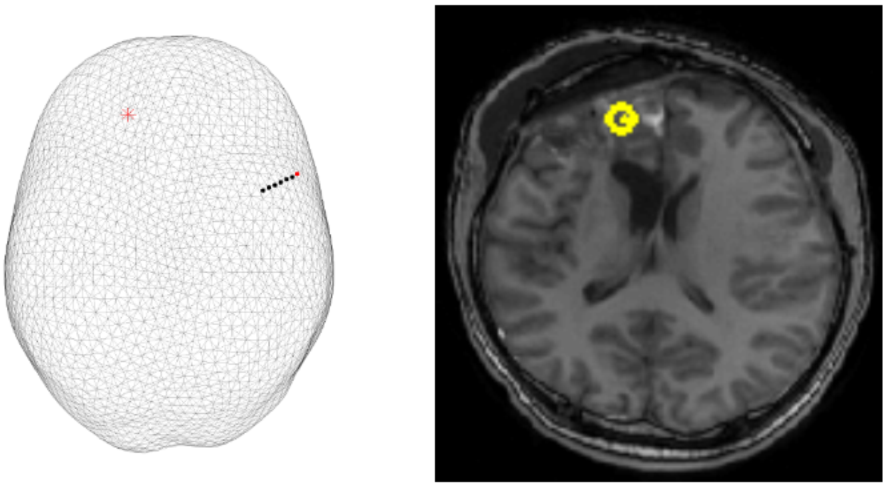

Figure 1. Difference in interictal source localisation between MEG dipole (red star) and SEEG peak amplitude channel (red circle), left, and MEG dipole on post-operative scan for that patient, right.

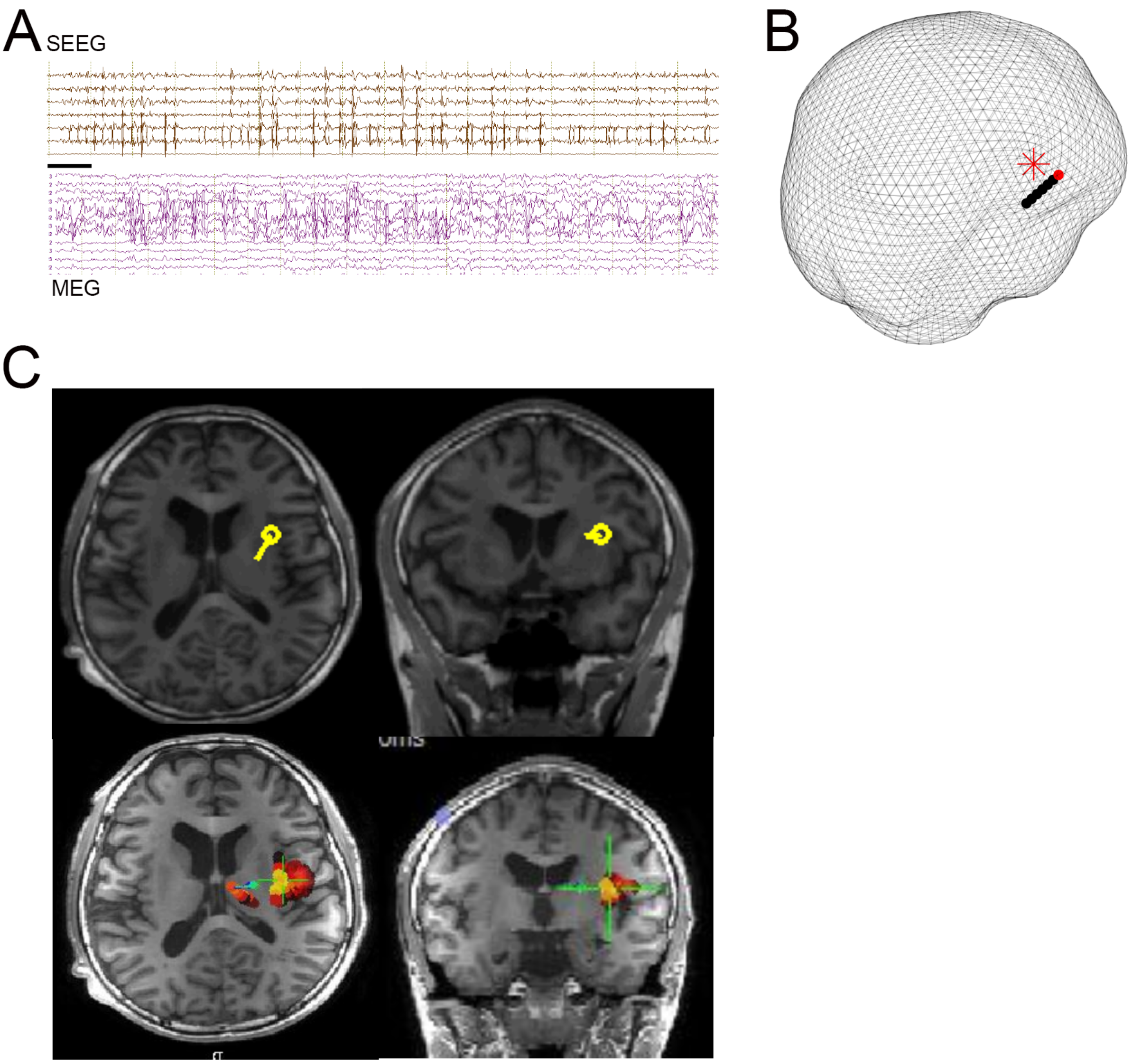

Figure 2. Seizure recorded in Patient 2 A. Raw SEEG (top) and MEG (bottom) traces Scale bar: 1s. B. Relation of MEG dipole and electrode contact with highest spike amplitude C. Position of MEG dipole based on interictal activity (upper) and beamformer based on ictal activity (lower).
